# Safety-First Framework for AI-Enabled Anamnesis in Head and Neck Surgery: Evidence Synthesis from a Narrative Review

**DOI:** 10.3390/jcm15062218

**Published:** 2026-03-14

**Authors:** Luigi Angelo Vaira, Hareem Qadeer, Jerome R. Lechien, Antonino Maniaci, Fabio Maglitto, Stefania Troise, Carlos M. Chiesa-Estomba, Giuseppe Consorti, Giulio Cirignaco, Giannicola Iannella, Carlos Navarro-Cuéllar, Giovanni Salzano, Giovanni Maria Soro, Paolo Boscolo-Rizzo, Valentino Vellone, Giacomo De Riu

**Affiliations:** 1 Maxillofacial Surgery Operative Unit, Department of Medicine, Surgery and Pharmacy, University of Sassari, 07100 Sassari, Italy; h.qadeerahmad@studenti.uniss.it (H.Q.); gderiu@uniss.it (G.D.R.); 2 PhD School of Biomedical Sciences, Department of Biomedical Science, University of Sassari, 07100 Sassari, Italy; 3 Department of Surgery, Mons School of Medicine, Research Institute for Health Sciences and Technology, University of Mons (UMons), 7000 Mons, Belgium; jerome.lechien@umons.ac.be; 4 Department of Otolaryngology-Head Neck Surgery, Elsan Polyclinic of Poitiers, 86000 Poitiers, France; 5 Department of Medicine and Surgery, University of Enna Kore, 94100 Enna, Italy; tnmaniaci29@gmail.com; 6 Head and Neck Section, Department of Neurosciences, Reproductive and Odontostomatological Science, Federico II University of Naples, 80131 Naples, Italy; fmaglitto@gmail.com (F.M.); stefy.troise@gmail.com (S.T.); giovannisalzanomd@gmail.com (G.S.); 7 Department of Otorhinolaryngology-Head & Neck Surgery, Hospital Universitario Donostia, 20014 San Sebastian, Spain; chiesaestomba86@gmail.com; 8 Division of Maxillofacial Surgery, Department of Neurological Sciences, Marche University Hospitals—Umberto I, 60121 Ancona, Italy; giuseppe.consorti@ospedaliriuniti.marche.it (G.C.); giuliocirignaco@gmail.com (G.C.); 9 Department of Biomedical Sciences and Public Health, Polytechnic University of Marche, 60121 Ancona, Italy; 10 Department of Medicine, Section of Maxillo-Facial Surgery, University of Siena, Viale Bracci, 53100 Siena, Italy; 11 Department of ‘Organi di Senso’, University “Sapienza”, Viale dell’Università, 33, 00185 Rome, Italy; giannicola.iannella@uniroma1.it; 12 Maxillofacial Surgery Department, Hospital Gregorio Marañon, Universidad Complutense de Madrid, 28040 Madrid, Spain; cnavarrocuellar@gmail.com; 13 Administrative Direction, University of Sassari, 07100 Sassari, Italy; gmsoro@uniss.it; 14 Department of Medical, Surgical and Health Sciences, Section of Otolaryngology, University of Trieste, 34127 Trieste, Italy; pboscolorizzo@yahoo.it; 15 Department of Life Science, Health, and Health Professions, Università degli Studi “Link”, 00165 Rome, Italy; v.vellone@unilink.it

**Keywords:** artificial intelligence, medical history taking, anamnesis, conversational AI, chatbots, large language models, symptom checker, digital triage, clinical decision support systems, head and neck surgery

## Abstract

**Objectives**: To synthesize evidence on artificial intelligence (AI)-enabled medical history taking (anamnesis)—beyond large language models (LLMs) alone—and to translate findings into implications and research priorities for head and neck surgery. **Methods**: We performed a PRISMA-informed narrative review. Searches from database inception to 31 December 2025 (updated 3 January 2026) were conducted in MEDLINE (PubMed), Embase, Scopus, Web of Science Core Collection, IEEE Xplore, and ACM Digital Library, supplemented by medRxiv/arXiv screening and citation chasing. We included studies evaluating or describing AI-supported history capture/summarization, conversational interviewing, symptom checker/digital triage, EHR-integrated intake-to-decision support pipelines, voice interviewing, education/training systems, and governance/ethical considerations related to digital anamnesis. Findings were synthesized by system category and by cross-cutting outcome domains, with a head and neck surgery interpretive lens. **Results**: Fifty studies (2014–2025) were included. Evidence most consistently suggested feasibility and acceptability of pre-consultation computer-assisted history taking and the potential to reduce documentation burden and improve structured capture. In contrast, symptom checkers and digital triage tools showed highly variable diagnostic/triage performance and prominent safety concerns, highlighting the importance of conservative red-flag escalation strategies, continuous monitoring, and clear accountability. LLM-based diagnostic dialogue demonstrated strong performance in controlled evaluations, but prospective real-world validation, governance, and workflow integration remain limited. **Conclusions**: AI-enabled anamnesis comprises heterogeneous tools with uneven evidence. For head and neck surgery, potential near-term applications may include structured pre-visit intake, clinician-facing summarization, and training applications, whereas autonomous triage warrants harm-oriented, specialty-calibrated validation and robust governance prior to broader clinical reliance.

## 1. Introduction

Medical history taking remains the cornerstone of clinical reasoning, risk stratification, and shared decision-making. Yet, the progressive shift from traditional face-to-face interviewing toward digital and semi-automated anamnesis raises new questions about data completeness, narrative fidelity, privacy, accountability, and the potential unintended consequences of “structuring” a patient’s story into machine-readable formats [1]. In parallel, the rising workload and time pressure across outpatient and acute-care settings have accelerated interest in tools that can collect, summarize, and operationalize patient-reported information before (or alongside) clinician encounters [2,3,4,5].

Importantly, the field has evolved beyond a narrow focus on large language models (LLMs). Contemporary “Artificial Intelligence (AI) for anamnesis” spans a spectrum of systems, including (i) computer-assisted history taking (CAHT) platforms that guide patients through adaptive questionnaires and generate clinician-ready summaries; (ii) symptom checkers and digital triage tools; (iii) conversational user interfaces (rule-based, ML-based, or LLM-based) that emulate interview-style dialogue; and (iv) downstream clinical decision support systems (CDSS) that leverage longitudinal electronic health record (EHR) histories to predict diagnoses or next-step actions [2,3,4,5]. Scoping and systematic reviews consistently highlight substantial heterogeneity in clinical settings, user interaction modalities, outcomes, and evaluation quality—underscoring that “AI anamnesis” is best understood as an ecosystem rather than a single technology class [2,3,4].

Real-world studies support the feasibility of pre-consultation history-taking tools that can standardize intake, reduce documentation burden, and improve visit preparedness. For example, a prospective outpatient pilot demonstrated high completion rates, acceptable completion times, and strong patient acceptance for a tablet-based CAHT system that generated editable narrative reports integrated into the practice management system [6]. Complementary work in primary care has emphasized usability and validity considerations, proposing structured evaluations of app-based anamnesis to determine how closely patient-entered histories align with clinician-led interviews and how interface design influences response accuracy and safety-critical disclosures [7].

At the same time, digital triage and symptom checker tools have attracted scrutiny because of their safety implications. A recent systematic review reported marked variability in diagnostic and triage performance across tools and scenarios, reinforcing the need for robust benchmarking, transparent validation, and clinically governed implementation rather than “plug-and-play” deployment [8]. Implementation research further suggests that adoption is not determined by algorithmic performance alone: qualitative evidence from primary care indicates that perceived usefulness, integration into workflows, clarity of responsibilities (especially for red flags), and interoperability constraints can decisively shape whether clinicians rely on the tool’s summaries or instead privilege the patient’s free-text narrative [9].

More technically integrated systems illustrate how patient-reported intake can become actionable clinical documentation and CDS. SmartTriage, a patient-facing pre-visit system, combines free-text “reason for visit” with EHR history to personalize question sequencing and generate documentation and decision support outputs (e.g., diagnosis and suggested orders) [10]. Downstream CDSS models trained on large-scale EHR data similarly demonstrate that diagnostic prediction can be operationalized at scale when uncertainty thresholds, workflow embedding, and clinician trust calibration are explicitly addressed [11]. Foundational work on longitudinal EHR representations (e.g., recurrent neural network approaches) further supports the concept that “patient history” is a highly informative signal for prediction—strengthening the rationale for high-quality, structured, and reusable anamnesis inputs [12].

LLM-driven systems have recently expanded the design space by enabling more naturalistic diagnostic dialogue and flexible summarization. In a Nature study, an LLM-based conversational diagnostic system (AMIE) achieved strong performance in a structured OSCE-like evaluation across multiple axes including information gathering, diagnostic accuracy, and communication quality, while still leaving open questions about real-world safety, multilingual equity, and deployment governance [13]. Complementary simulated evaluations in emergency medicine have compared ChatGPT with physicians on clinical performance and communication, again emphasizing that conversational fluency does not automatically translate into safe clinical integration [14]. Earlier work on conversational user interfaces for self-anamnesis provides additional historical context: “conversational” interaction has long been pursued with rule-based methods, and many of the same usability and interpretation challenges remain relevant even as LLMs raise the ceiling of language understanding [15].

For head and neck surgery, this broader AI anamnesis landscape is highly relevant even if most published evaluations arise from other specialties. Head and neck patients frequently present with symptom constellations where subtle temporal patterns and “red-flag” combinations (e.g., persistent dysphonia, progressive dysphagia/odynophagia, neck mass, referred otalgia, weight loss, bleeding) critically influence urgency, diagnostic pathways, and multidisciplinary coordination. Moreover, perioperative trajectories often involve complex longitudinal histories (oncologic treatment, reconstruction, airway/swallow function, complications, adjuvant therapies) where structured, high-fidelity anamnesis could improve continuity across settings and reduce redundant questioning [16,17,18]. Conceptual frameworks that treat anamnesis as a multimodal, shareable “hub” for diagnostic reasoning—particularly in complex or rare disease contexts—suggest how similar architectures could be adapted for high-risk head and neck pathways, integrating patient-reported histories with imaging, reports, and specialist observations [19].

Against this background, the present narrative review synthesizes evidence on AI-enabled medical history taking across rule-based systems, ML-driven triage and intake platforms, and LLM-based conversational agents. We emphasize (i) the range of interaction modalities and outputs; (ii) the outcomes used to evaluate these tools (data quality, triage/diagnostic accuracy, safety, efficiency, acceptability, equity); and (iii) implementation requirements such as interoperability, human-in-the-loop design, and governance. Finally, we propose a specialty-facing interpretation of how these technologies could translate to head and neck surgery—identifying near-term use cases and research priorities needed for safe, clinically meaningful adoption.

## 2. Materials and Methods

This narrative review synthesizes evidence on AI-enabled medical history taking (anamnesis), spanning CAHT, digital triage and symptom checkers, conversational systems (rule-based, machine learning-based, and large language model-based), and selected downstream clinical decision support approaches that explicitly leverage patient history. To maximize transparency and reproducibility of the literature identification and reporting, the review was written in accordance with the PRISMA 2020 reporting framework where applicable and the PRISMA-S extension for reporting literature searches, acknowledging that some PRISMA elements are primarily designed for systematic reviews but can improve clarity when narrative reviews incorporate structured search methods [20,21].

Although a structured multi-database search and PRISMA-informed reporting were employed to enhance transparency and reproducibility, this work was conducted as a structured narrative review rather than a formal systematic review. Given the substantial heterogeneity of study designs (including prospective validation studies, vignette-based simulations, qualitative implementation research, and conceptual analyses), a formal risk-of-bias assessment or quantitative grading framework was not applied. Instead, studies were synthesized narratively across methodological categories to identify patterns, gaps, and safety-relevant considerations.

A comprehensive literature search was conducted from database inception to 20 December 2025. We searched the following databases to capture both biomedical and informatics/engineering outputs: MEDLINE (via PubMed), Embase, Scopus, Web of Science Core Collection, IEEE Xplore, and the ACM Digital Library. Because relevant work in conversational AI and clinical ML is frequently disseminated via conference proceedings and preprints, we additionally screened medRxiv and arXiv, and performed citation chasing (backward reference screening and forward citation tracking) of key eligible articles and high-yield reviews. They were excluded from the formal synthesis; however, selected conference publications were cited in the background where relevant to contextualize technical developments. The search strategy combined controlled vocabulary (when available) and free-text terms for (i) medical history taking and intake (e.g., “medical history taking,” “anamnesis,” “computer-assisted history taking,” “digital intake,” “pre-consultation”), and (ii) AI modalities and tools (e.g., “artificial intelligence,” “machine learning,” “natural language processing,” “chatbot,” “conversational AI,” “large language model,” “symptom checker,” “digital triage”). To support the head and neck surgery interpretive lens, additional terms were incorporated to capture otolaryngology–head and neck contexts (e.g., “maxillofacial-surgery”, “head and neck,” “otolaryngology,” “ENT,” “oral surgery”) when relevant. Importantly, eligibility was not restricted to head and neck specific populations; broader AI enabled anamnesis literature across clinical domains was included and subsequently interpreted through a head and neck surgery safety lens. Full search strings for each database are provided in Supplementary Table S1.

Eligibility criteria were defined a priori. We included peer-reviewed articles, and selected preprints that described or evaluated AI-enabled systems supporting history capture, interactive symptom elicitation, triage, clinician-ready summarization, or training in history taking, as well as evidence syntheses and ethical/legal analyses directly relevant to digital anamnesis and conversational systems. We also included foundational studies demonstrating how longitudinal patient history representations in EHRs contribute to prediction or decision support when conceptually tied to the value of structured anamnesis inputs. We excluded studies focused solely on unrelated AI applications without a history-taking component, conference abstracts lacking sufficient methodological detail to interpret the intervention or framework, and publications not pertinent to healthcare anamnesis.

Records were screened for relevance at the title/abstract level, followed by full-text assessment for inclusion. Data were extracted using a standardized form capturing publication characteristics, clinical setting, interaction modality (adaptive questionnaire, conversational chat, voice interview, hybrid), underlying computational approach (rule-based/knowledge-based, classical ML, transformer-based NLP, LLM, or hybrid), inputs (free text, structured responses, EHR history, demographics), outputs (summary/note, triage recommendation, differential diagnosis, suggested orders, educational feedback), degree of workflow/EHR integration, and outcomes (data quality/completeness, diagnostic/triage accuracy, safety/red-flag handling, efficiency/documentation burden, acceptability, and equity considerations). Evidence was synthesized narratively using a dual organization: first by system category (pre-consultation CAHT, triage/symptom checkers, integrated intake-to-CDS pipelines, LLM diagnostic dialogue, voice interviewing, education/training), and second by cross-cutting outcome domains to enable comparison across heterogeneous designs. To improve interpretability across heterogeneous designs, studies were additionally considered according to validation context (prospective real-world evaluation, retrospective dataset validation, vignette-based simulation, qualitative implementation research, or conceptual analysis). Finally, findings were interpreted through a head and neck surgery lens, focusing on translational implications for safety-critical symptom patterns, escalation pathways, multidisciplinary coordination, and longitudinal perioperative care trajectories.

## 3. Results

### 3.1. Study Selection

**Figure 1:**
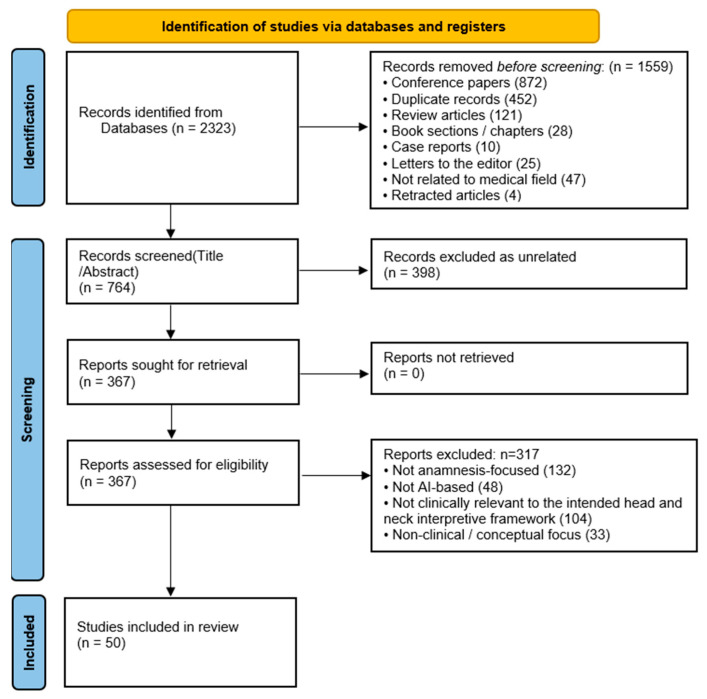
PRISMA flow diagram of the study selection process.

The database searches identified 2323 records. Prior to screening, 1559 records were removed, including 872 conference abstracts without sufficient methodological detail, 452 duplicates, 121 review articles, 28 book chapters, 10 case reports, 25 letters to the editor, 47 records not related to the medical field, and 4 retracted articles. A total of 764 records were screened at the title and abstract level, of which 398 were excluded for being unrelated. Full-text assessment was conducted for 367 articles, and 317 were excluded after review (132 not focused on anamnesis, 48 not AI-based, 104 not clinically relevant to the intended head and neck interpretive framework, and 33 non-clinical or primarily conceptual). Ultimately, 50 studies were included in the narrative synthesis [Figure 1] [6,7,8,10,11,13,14,15,19,22,23,24,25,26,27,28,29,30,31,32,33,34,35,36,37,38,39,40,41,42,43,44,45,46,47,48,49,50,51,52,53,54,55,56]. Study characteristics and specialty-facing notes are summarized in Supplementary Tables S1 and S2.

#### Characteristics of Included Studies

The included evidence spanned 2014–2025 and was methodologically heterogeneous, comprising clinical pilots and observational evaluations in real patients and real workflows (including outpatient/urgent care and emergency department contexts) [6,30,33,41,51], vignette-based benchmarking and validation studies of triage and diagnostic outputs [9,29,32,34,54,56], qualitative implementation research focused on adoption, workflow fit, and accountability [8], randomized or pilot comparative designs assessing history-taking devices and downstream effects [25], study protocols designed to evaluate usability and validity of digital anamnesis tools [7], technical and methodological ML studies centered on question selection, re-ranking, representation learning, endpoint construction, and digitization/standardization of history [10,11,12,22,24,31,40,47,53], and multiple scoping/systematic reviews and broader syntheses mapping the landscape of digital history taking, chatbots, and triage systems [2,3,4,5,28]. Ethical and medico-legal analyses specifically addressing the implications of shifting from traditional to digital anamnesis were also included [1], together with conceptual or framework-oriented contributions relevant to anamnesis modernization and integration with multimodal diagnostic reasoning [19,52].

Across the corpus, most primary evaluations were performed in general medicine, primary care, urgent care, emergency medicine, and cardiology, reflecting where intake and triage bottlenecks have been most actively targeted [4,6,7,8,9,30,33,41]. Only a minority of papers were directly situated within otolaryngology/head and neck practice, Accordingly, specialty relevance was frequently inferred from generalizable mechanisms (such as red-flag detection, workflow integration, data quality, documentation burden, and communication support) rather than from head and neck-specific prospective validation [1,8,9,41]. Nevertheless, several head-and-neck-adjacent or maxillofacial/ENT-relevant contributions were identified, providing partial contextual support for cautious specialty translation [16,17,18].

### 3.2. Findings by System Category

#### 3.2.1. Pre-Consultation CAHT and Digital Intake Tools

Real-world prospective pilot studies generally supported the feasibility of pre-consultation intake systems, reporting high completion rates, acceptable completion times, and favorable patient acceptance in outpatient contexts [6,30]. A large prospective pilot in outpatient care demonstrated that tablet-based, dynamically guided history taking could produce editable narrative reports suited for clinical use and integrated into practice workflows, with iterative refinement improving report quality over time [6]. In primary care, protocol-driven work emphasized that digital anamnesis should be evaluated not only for usability but also for concurrent validity against clinician-led interviews, because interface design and question framing can affect whether safety-critical information is correctly elicited and recorded [7]. A broader review-level perspective similarly concluded that CAHT is typically acceptable and operationally feasible, but emphasized substantial heterogeneity in endpoints and evaluation designs, with limited prospective evidence demonstrating consistent effects on hard clinical outcomes [4,5]. Evidence is also emerging that “automatic history-taking software” can affect measurable aspects of data quality in outpatient specialty workflows, reinforcing that digital intake can influence the content and structure of clinical information beyond simple convenience [33]. Pilot randomized work testing automated history-taking devices in ambulatory care further suggests that these tools can be evaluated in comparative designs, though generalizability and endpoint standardization remain challenges [25].

#### 3.2.2. Digital Triage and Symptom Checker Systems

Across systematic reviews and primary benchmarking studies, the diagnostic and triage performance of symptom checkers appeared highly variable, strongly dependent on the tool, clinical scenario, and evaluation method [9,29,54,56]. Longitudinal evaluations suggested that performance can change over time, supporting the need for continuous monitoring and re-validation as models and product versions evolve [32]. Importantly, a validation strategy anchored to historical triage-related adverse events offered a clinically meaningful way to test triage safety beyond headline accuracy metrics, directly addressing the failure modes most relevant to patient harm [34]. In specialty-oriented experimental comparisons, AI chatbot performance for diagnostic reasoning has been shown to lag behind expert assessment in certain domains, underscoring that “conversational plausibility” is not equivalent to clinical correctness and that benchmarking must be context-specific [54]. Recent interactive vignette-based work also expanded evaluation beyond endpoint accuracy by assessing the history-elicitation process (e.g., adaptive questioning behavior and capture of clinically relevant features), providing a potentially transferable method for specialty-specific tool assessment [56]. Collectively, these findings suggest that triage tools may offer workflow value; their use in high-risk presentations should be accompanied by clearly defined safety thresholds, validated escalation pathways, and specialty-calibrated evaluation [1,8,9,34].

#### 3.2.3. EHR-Integrated Intake-to-Documentation Pipelines and Downstream CDS Anchored in Patient History

Multiple studies demonstrated how patient-entered or history-derived information can be operationalized into documentation and decision support when linked to EHR context. SmartTriage illustrated a scalable approach in which a free-text “reason for visit” is mapped to structured complaints, adaptive question sequencing is personalized using patient history, and downstream models generate diagnostic and order-related outputs; performance improved when longitudinal history was incorporated, particularly in populations with chronic disease burden [10]. A large EHR-based diagnostic assistant (AIDA) similarly operationalized diagnostic prediction at scale and explicitly introduced uncertainty gating (showing output only above confidence thresholds), underscoring the practical importance of workflow integration and trust calibration [11]. Foundational modeling work on representing longitudinal EHR histories supported the broader rationale that “patient history” is a high-value predictive signal, which supports the conceptual rationale for capturing anamnesis in structured, reusable formats [12,24]. Methodological analyses further cautioned that definitions of clinical endpoints and the modality used to derive them (codes, notes, labs, etc.) can materially alter model behavior and conclusions, highlighting a key translational issue when building CDSS from heterogeneous clinical data streams [40]. Additional work on history classification and feature construction reinforced that the way history is structured and represented directly affects downstream ML performance, again emphasizing that “anamnesis quality” is not only a clinical issue but a computational one [47]. Finally, efforts aimed at digitizing medical histories into standard formats suggest a pathway toward interoperability and reuse across settings—particularly relevant for multidisciplinary care trajectories [31].

#### 3.2.4. Conversational Interviewing Systems and LLM-Based Diagnostic Dialogue

Conversational approaches ranged from early rule-based self-anamnesis systems to contemporary LLM-driven diagnostic dialogue tools. Rule-based conversational self-anamnesis demonstrated the feasibility of dialogue-style intake and highlighted persistent challenges related to handling user input variability and maintaining clinical completeness within constrained dialogue flows [15]. In clinical environments, an emergency department mixed-methods pilot of an AI symptom-taking tool suggested that structured symptom capture may support aspects of the patient–physician conversation and information organization, although endpoints were primarily feasibility and acceptability rather than definitive clinical outcomes [41]. In controlled comparative evaluations, LLM-based systems designed for diagnostic dialogue demonstrated strong performance across information gathering, diagnostic reasoning, and communication domains in OSCE-like assessments, but these findings remain limited by simulated settings and unresolved questions about real-world safety, multilingual equity, and governance [13]. Complementary simulated comparisons of ChatGPT versus physicians similarly indicated potential strengths in communication and history-taking completeness, while underscoring the gap between conversational fluency and safe clinical integration [14]. Technical work on dialogue-contextualized re-ranking proposed mechanisms to improve question selection during history taking based on conversational context, offering a methodological bridge between rigid questionnaires and more adaptive interviewing systems [53]. In contrast, a set of general “disease prediction chatbot” and “digital health partner” papers typically proposed architectures with limited rigorous validation of anamnesis quality, safety/escalation handling, or real-world effectiveness [42,43,44,48].

#### 3.2.5. Voice-Based Interviewing

Evidence for voice interviewing remains early-stage. A prototype system explored voice-based interviewing for diagnostic support, suggesting feasibility of spoken interaction for history capture but providing limited clinical validation and uncertain generalizability [39]. This modality may be relevant for accessibility (e.g., low literacy, reduced typing ability), but robust evaluations in real workflows are still scarce [4,39].

#### 3.2.6. Education and Training (Virtual Patients, Role-Play, and Automated Feedback)

A substantial portion of recent work examined history-taking training using simulated patients, role play, and automated feedback. Survey and feasibility studies suggested that AI-based role plays can provide teaching opportunities and are generally acceptable to learners [35]. Multiple systems integrated LLM-based agents into virtual patients or simulated patient frameworks, often reporting feasibility, user engagement, and the ability to generate structured feedback on interview performance [36,37,38,45,46,49,50]. Importantly, these studies also documented failure modes relevant to clinical translation, such as variability in automated feedback quality depending on rubric design, and risks of plausible but incorrect content generation when models are pushed beyond constrained scenarios [45,46,50]. Collectively, education-focused evidence indicates that structured rehearsal and assessment of head-and-neck-relevant symptom interviews may represent a potentially adaptable application, provided that governance and evaluation rubrics are carefully designed [35,36,37,38,45,46,49,50].

#### 3.2.7. Synthesis Papers and Governance-Focused Literature

Scoping and systematic reviews consistently described a field dominated by prototypes, vignette-based evaluations, and heterogeneous endpoints, with relatively limited evidence for prospective integration, standardized outcome reporting, and robust assessment of safety and equity [2,3,4,5,28]. Ethical and medico-legal analyses emphasized tensions between efficiency gains and risks to narrative fidelity, privacy, equity, and accountability—considerations that become particularly salient when AI tools are positioned upstream of urgent referral decisions or oncologic pathways [1]. Complementary guidance and conceptual work framed the diagnostic value of patient history as a data modality and provided practical lessons for designing “intelligent interviewers,” highlighting the potential value of high-fidelity anamnesis capture and careful human-in-the-loop implementation [5,23,26,27,52]. Framework-oriented proposals also argued for multimodal, shareable anamnesis “hubs” to support diagnostic reasoning in complex contexts, a concept that may be adaptable high-risk head and neck pathways [19].

### 3.3. Cross-Cutting Outcomes Across Studies

#### 3.3.1. Data Quality and Completeness

Evidence on data quality was most directly addressed by CAHT implementations and outpatient workflow studies where structured capture and narrative report generation were feasible and associated with perceived or measurable improvements in documentation and data completeness [4,6,33,41]. In addition, NLP methods enabling extraction of family history from clinical notes demonstrated a pathway to reduce loss of hereditary risk information embedded in unstructured documentation [22]. However, across reviews, definitions of “completeness” and “quality” remained inconsistent, limiting cross-study comparability and precluding pooled estimates [4,5,28].

#### 3.3.2. Diagnostic Accuracy and Triage Performance

Across symptom checkers, diagnostic accuracy for the top-ranked diagnosis was frequently limited and variable, while triage performance showed broader dispersion across tools and scenarios [9,29,32,34,54,56]. Longitudinal and safety-anchored evaluations highlighted the importance of monitoring tool evolution and validating against clinically meaningful harm-oriented scenarios rather than accuracy alone [32,34,56]. In controlled settings, conversational diagnostic systems and EHR-based CDSS models reported strong performance; however, translation to real-world practice requires prospective integration studies, explicit escalation design, and governance mechanisms [11,13,14,40].

#### 3.3.3. Safety and Red-Flag Handling

Across multiple evaluations and implementation analyses, safety concerns were frequently highlighted, particularly the risk of false reassurance, unclear escalation responsibility, and the mismatch between algorithmic output and real-world accountability [1,4,8,9,34,56]. Implementation-focused evidence indicated that even when tools appear promising, adoption and safe use are strongly shaped by workflow embedding, clarity of ownership for red flags, and interoperability constraints that affect how reliably clinicians can act on the captured history [4,8,41].

#### 3.3.4. Efficiency, Documentation Burden, and Workflow Integration

The most consistent process-level signals related to feasibility and documentation support: CAHT and integrated intake systems could be completed within acceptable time windows and generate clinician-facing narratives or structured summaries, with reported or perceived impacts on documentation workload [4,6,10,33]. However, reviews noted variable effects on consultation length and emphasized that workflow benefits depend on integration depth and local implementation strategy [4,5].

#### 3.3.5. Acceptability and User Experience

In CAHT pilots and feasibility studies of conversational interviewing systems, patient acceptance was generally reported as favorable, although contexts and measurement approaches varied widely [4,6,30,41,51]. Reviews focused on chatbots and digital history-taking tools similarly suggested generally positive acceptability signals, but also highlighted limited standardization of user-centered endpoints and a shortage of long-term follow-up data [3,4,5].

#### 3.3.6. Equity and Implementation Constraints

Equity concerns—including digital literacy, language barriers, access, and differential capability to provide accurate histories through digital interfaces—were emphasized in implementation studies and governance-oriented literature and were repeatedly identified as unresolved barriers in broader reviews [1,4,5,8]. These considerations are particularly relevant when extrapolated to head and neck pathways, where delayed escalation or incomplete red-flag histories may carry disproportionate consequences [1,8,34].

## 4. Discussion

This narrative synthesis shows that “AI for anamnesis” is not a single intervention but a continuum of tools that differ in interaction modality, technical approach, workflow position, and safety profile. Across scoping and systematic reviews, the dominant pattern is methodological heterogeneity and a persistent gap between prototype performance and evidence of real-world clinical benefit, particularly in high-stakes settings where escalation decisions matter most [2,3,4,5]. At the same time, multiple strands of evidence suggest that: improving the capture, structure, and reusability of patient history may reduce documentation burden, enhance continuity, and potential implications for decision-making—provided that implementation is governed, human-in-the-loop, and safety-centered rather than technology-centered [1,4,6,10,11].

### 4.1. From “Chatbots” to an Ecosystem: Why Broad AI (Not Only LLMs) Is the Correct Framing

A key finding is that the most implementation-ready benefits to date often arise from systems that are not LLM-based at all. Computer-assisted history taking (CAHT) and app-based digital intake tools can standardize pre-consultation capture and generate clinician-editable narratives, with feasibility and acceptance signals in real outpatient workflows and emerging evidence on documentation/data quality effects [4,5,6,7,30,33]. In parallel, more “AI-intensive” pipelines that couple patient-entered intake with EHR history demonstrate the technical feasibility of personalization (adaptive question sequencing) and downstream decision support, with consistent indications that longitudinal history adds value—especially in patients with chronic disease and complex trajectories [10,11,12,24]. Together, these findings suggest that AI enabled anamnesis may be more conceptualized as an ecosystem spanning structured intake, adaptive interviewing, summarization, and downstream CDS rather than equated solely with LLM based chat system [2,3,4,5,10,11,12].

### 4.2. Safety Is the Central Challenge—Especially for Triage and Red Flags

The current evidence base frequently highlights safety as a central consideration for broader deployment, particularly for symptom checkers and digital triage tools. Systematic review evidence shows substantial variability in diagnostic and triage accuracy across tools and scenarios and highlights the frequent reliance on vignette-based evaluations, which may underestimate real-world failure modes [9]. Longitudinal follow-up studies reinforce that performance can drift as products evolve, supporting the need for continuous validation rather than one-time certification [32]. Importantly, approaches anchored to real-world adverse events or harm-relevant vignette sets offer a more clinically aligned validation pathway than accuracy alone [34]. Newer interactive evaluation designs that explicitly assess the history-elicitation process (what was asked, what was captured) are particularly relevant to specialty practice because they make safety and completeness measurable upstream of the “final answer” [56]. In head and neck surgery, where delays in escalation for dysphagia, dysphonia, neck mass, bleeding, or weight loss can change prognosis, these findings suggest that the use of generic symptom checkers as gatekeepers in high-risk head and neck presentations warrants caution unless accompanied by specialty-calibrated red-flag logic, clearly defined escalation thresholds, and explicit accountability pathways [1,9,34,56].

### 4.3. Workflow Integration Determines Whether “Good AI” Becomes Useful AI

Implementation research shows that adoption is not determined by algorithmic performance alone. Qualitative evidence from primary care highlights that clinician trust, clarity of responsibility for red flags, and interoperability constraints shape whether AI outputs are used or ignored, with clinicians often privileging the patient’s free-text narrative over AI summaries when integration is poor or accountability is ambiguous [8]. Similar lessons emerge from feasibility studies of symptom-taking tools in emergency care, where perceived value lies in organizing information and supporting the conversation, but hard outcomes and longitudinal governance remain underdeveloped [4,41]. In this context, confidence gating and selective display of outputs—demonstrated in deployed EHR-based diagnostic assistants—illustrate a pragmatic safety pattern in which support is surfaced selectively, particularly when uncertainty is acceptably low and when clinicians can interrogate and override the output [11]. In head and neck workflows, these may support an approach in which AI intake functions primarily as an upstream preparation layer (capturing key features, exposures, timelines, and red flags) with diagnostic or triage decisions remaining clinician-mediated, particularly until specialty-specific prospective evidence becomes available [4,6,8,11].

### 4.4. LLMs Raise the Ceiling of Interaction—But Do Not Remove the Need for Governance

LLM-based diagnostic dialogue systems show impressive controlled performance on history taking, reasoning, and communication in OSCE-like evaluations, suggesting that LLMs can act as powerful “information acquisition engines” when prompts, rubrics, and contexts are constrained [13]. Simulated comparisons in emergency medicine similarly suggest strengths in communication and completeness, but also highlight that conversational fluency can mask uncertainty and does not guarantee safe real-world behavior [14]. Earlier rule-based conversational self-anamnesis work is a useful reminder that many core challenges—clarifying ambiguous input, maintaining coverage without excessive burden, and supporting usability across diverse patients—predate LLMs and will persist even as language understanding improves [15]. Technical advances such as dialogue-contextualized re-ranking point toward more principled adaptive questioning, but clinical translation will depend on how these methods are validated against safety-relevant endpoints and integrated into workflows with clear escalation logic [53]. In head and neck practice, LLMs may be most appropriately explored as (i) structured summarizers of pre-visit histories, (ii) interview assistants that ensure coverage of standardized red-flag and exposure domains, and (iii) patient-facing educational supports—while their use as autonomous triage agents remains insufficiently validated in specialty-specific contexts [1,13,14,15].

### 4.5. Preserving the Patient Story While Structuring the Data: A Head-and-Neck-Relevant Tension

Ethical and legal analyses emphasize that digitizing anamnesis risks degrading the patient’s narrative, shifting ownership of the story, and introducing new privacy and accountability vulnerabilities [1]. This is particularly salient in head and neck oncology and functional disorders (speech, swallowing, pain, appearance), where psychosocial context and symptom nuance strongly influence assessment, shared decision-making, and adherence. In head and neck contexts, implementation would likely need to balance two goals: structuring key clinical variables for safety and continuity while preserving free narrative that captures nuance, priorities, and lived experience [1,8]. From a design standpoint, hybrid intake that combines adaptive structured questions with protected free-text narrative fields—and produces summaries that explicitly separate “patient-reported narrative” from “system-extracted structured features”—may represent one potential approach to reducing information loss and medico-legal ambiguity [1,6,8].

### 4.6. Translating the Evidence to Head and Neck Surgery: Practical Near-Term Use Cases

**Figure 2:**
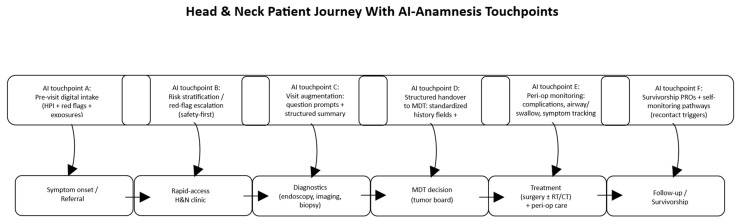
Head and neck patient journey with integrated AI-assisted anamnesis touchpoints. The diagram depicts the main stages of the patient pathway—from symptom onset to follow-up and survivorship—highlighting where AI can support the process.

Although most included studies originate outside head and neck care, several potential translational use cases can be considered. First, pre-consultation intake for rapid-access head and neck clinics may help standardize capture of red flags and timeline features and reduce redundant questioning, especially for high-volume referral pathways; CAHT feasibility and documentation outputs provide the closest real-world analogs [4,6,7,30]. Second, perioperative and survivorship longitudinal history capture may support continuity across multidisciplinary care (surgery, radiation oncology, medical oncology, speech-language pathology), where symptom trajectories and complications evolve over time; the value of longitudinal history representations in EHR-based models supports the concept that structured histories can be computationally and clinically useful downstream [12,24,40]. Third, specialty-tailored education and training represents a potentially near-term application area: LLM-based simulated patients and automated feedback systems are already being tested and can be adapted to head-and-neck-specific interview patterns (e.g., neck mass workup, dysphagia history, airway risk, oral lesion risk factors), with governance focused on rubric validity and feedback reliability [35,36,37,38,45,46,49,50]. Finally, head-and-neck-adjacent studies—such as diagnostic performance assessments of multimodal AI in oral mucosal lesions or ENT-oriented AI applications—indicate that the specialty is already engaging with AI in adjacent domains, suggesting that further consideration of anamnesis and workflow integration may be warranted [16,17,18] [Figure 2].

### 4.7. How Head and Neck Should Evaluate These Tools: Beyond Endpoint Accuracy

A recurring limitation in the literature is the mismatch between what is measured and what matters clinically. For head and neck pathways, evaluation may benefit from going beyond “top-1 diagnosis accuracy” and include (i) capture of predefined red-flag features and exposures, (ii) correctness of timeline representation, (iii) escalation recommendations under uncertainty, and (iv) auditability of summaries. In addition, ENT-specific instruments designed to assess chatbot performance may contribute to more standardized evaluations and reporting in specialty contexts [17]. Framework-oriented proposals that treat anamnesis as a multimodal hub further outline a possible long-term direction: integrating patient narrative, structured symptoms, images, and clinician hypotheses in a governed architecture—an approach that may be valuable for complex head and neck presentations that traverse multiple disciplines and diagnostic modalities [19].

### 4.8. Limitations of the Evidence and of This Review

This review reflects a literature characterized by heterogeneity in settings, endpoints, and evaluation rigor, with frequent reliance on simulated vignettes and limited prospective evidence of patient-level outcomes [1,2,3,4,5,9]. Many “chatbot” papers propose architectures without robust validation of safety, escalation handling, or workflow effectiveness, limiting generalizability to clinical adoption decisions [42,43,44,48]. Even for stronger studies, transferability to head and neck surgery remains inferential because few evaluations are specialty-specific [1,4,8]. As a narrative review, our synthesis emphasizes conceptual integration and translational interpretation rather than pooled effect estimation, and conclusions should therefore be considered hypothesis-generating with respect to specialty implementation priorities [2,3,4,5].

### 4.9. Research Agenda for Head and Neck Surgery

The most urgent specialty research needs are pragmatic and safety-oriented. Prospective evaluations in head and neck clinics could examine whether AI-supported anamnesis improves completeness of red-flag capture, reduces time-to-decision, and improves documentation quality without increasing false reassurance or widening inequities. Such studies may incorporate harm-relevant scenarios and explicit escalation pathways, evaluate multilingual and low-literacy performance, and report governance measures such as uncertainty gating, audit trails, and clinician override rates [1,4,8,11,13,34,56]. Parallel educational trials could develop validated rubrics for head-and-neck-specific history taking and evaluate whether simulated-patient tools improve learner performance and reduce variability in interviewing quality [35,36,37,38,45,46,50]. Finally, interoperability and standardization efforts—digitizing histories in reusable formats and aligning capture to downstream CDS needs—may warrant exploration in multidisciplinary head and neck trajectories where continuity is a known pain point [4,31,40].

## 5. Conclusions

Overall, existing evidence suggests the feasibility and potential utility of AI-enabled anamnesis, but it also shows that safety, governance, and workflow integration are decisive for clinical value. For head and neck surgery, near-term applications may most reasonably focus on structured pre-visit capture, clinician-facing summarization, and education/training, while autonomous triage warrants cautious evaluation and validated against harm-oriented, specialty-relevant endpoints. The field may be transitioning from general claims about “AI chatbots” toward more specialty-calibrated, workflow-embedded, safety-oriented implementations that preserve the patient story while improving the reliability and reuse of clinical history data.

## Data Availability

No new data were created or analyzed in this study. Data sharing is therefore not applicable to this article. All data supporting the findings of this narrative review are contained within the article itself and in the publicly available studies cited in the reference list.

## Supplementary Materials

The following supporting information can be downloaded at: https://www.mdpi.com/article/10.3390/jcm15062218/s1, Table S1: Database-specific search strategies; Table S2: Characteristics of included studies and head & neck relevance mapping.
