# Supplementary material for: Safety-First Framework for AI-Enabled Anamnesis in Head and Neck Surgery: Evidence Synthesis from a Narrative Review
Source: J Clin Med. 2026 Mar 14;15(6):2218. doi: 10.3390/jcm15062218 (PMC13026613; doi:10.3390/jcm15062218)
Supplement: Supplementary file 1 [file jcm-15-02218-s001.zip › jcm-4180521-supplementary.pdf]

**Supplementary Table S1. Database-specific search strategies.**

| Database / Platform | Search string (as executed; adapted syntax per database) | Limits / Notes |
|---|---|---|
| PubMed (MEDLINE) | (("medical history taking"[Title/Abstract] OR "history taking"[Title/Abstract] OR anamnesis[Title/Abstract] OR "computer-assisted history"[Title/Abstract] OR "computer assisted history"[Title/Abstract] OR "digital intake"[Title/Abstract] OR "pre-consultation"[Title/Abstract] OR "preconsultation"[Title/Abstract] OR "symptom checker"[Title/Abstract] OR "digital triage"[Title/Abstract]) AND ("artificial intelligence"[Title/Abstract] OR "machine learning"[Title/Abstract] OR "deep learning"[Title/Abstract] OR "natural language processing"[Title/Abstract] OR NLP[Title/Abstract] OR chatbot*[Title/Abstract] OR "conversational AI"[Title/Abstract] OR "large language model"[Title/Abstract] OR LLM[Title/Abstract])) OR (("medical history taking"[MeSH Terms] OR "patient history"[Title/Abstract]) AND ("artificial intelligence"[MeSH Terms] OR "machine learning"[Title/Abstract] OR chatbot*[Title/Abstract])) | Humans; no language restriction at search stage; filters applied during screening |
| Embase | ('medical history taking':ti,ab OR 'history taking':ti,ab OR anamnesis:ti,ab OR 'computer assisted history':ti,ab OR 'digital intake':ti,ab OR preconsultation:ti,ab OR 'symptom checker':ti,ab OR 'digital triage':ti,ab) AND ('artificial intelligence':ti,ab OR 'machine learning':ti,ab OR 'deep learning':ti,ab OR 'natural language processing':ti,ab OR chatbot*:ti,ab OR 'conversational ai':ti,ab OR 'large language model':ti,ab OR llm:ti,ab) | Emtree terms exploded where available; duplicates removed against PubMed set |
| Scopus | TITLE-ABS-KEY(("medical history taking" OR "history taking" OR anamnesis OR "computer-assisted history" OR "digital intake" OR preconsultation OR "symptom checker" OR "digital triage") AND ("artificial intelligence" OR "machine learning" OR "deep learning" OR "natural language processing" OR chatbot* OR "conversational ai" OR "large language model" OR llm)) | Used for forward citation tracking of key studies and reviews |
| Web of Science Core Collection | TS=(("medical history taking" OR "history taking" OR anamnesis OR "computer-assisted history" OR "digital intake" OR preconsultation OR "symptom checker" OR "digital triage") AND ("artificial intelligence" OR "machine learning" OR "deep learning" OR "natural language processing" OR chatbot* OR "conversational ai" OR "large language model" OR llm)) | Used to capture interdisciplinary and health services literature not indexed elsewhere |
| IEEE Xplore | ("medical history" OR "history taking" OR anamnesis OR "symptom checker" OR triage) AND ("artificial intelligence" OR "machine learning" OR "natural language processing" OR chatbot OR "conversational" OR "large language model") | Focus on engineering/informatics implementations; conference proceedings included |
| ACM Digital Library | (("medical history" OR "history taking" OR anamnesis OR "symptom checker" OR triage) AND ("artificial intelligence" OR "machine learning" OR "natural language processing" OR chatbot OR "conversational" OR "large language model")) | Focus on HCI and conversational agent design relevant to history taking |
| medRxiv | ("symptom checker" OR "digital triage" OR "medical history taking" OR anamnesis OR chatbot OR "conversational AI" OR "large language model") | Preprints screened when providing unique evaluation methods/results |
| arXiv | ("medical history" OR "history taking" OR anamnesis OR triage OR "symptom checker") AND (chatbot OR "conversational" OR "large language model" OR "machine learning") | Used to identify early technical reports subsequently cross-checked for peer-reviewed versions |

**Supplementary Table S2. Characteristics of included studies and head & neck relevance mapping.**

| Author, year (Ref) | Clinical context / population | System & AI approach (single-cell summary) | Study design & outcomes assessed | Main findings | Key limitations / notes | Potential relevance to head & neck surgery |
|---|---|---|---|---|---|---|
| Bill, 2014 [22] | Clinical notes (EHR free text) | NLP pipeline to extract family history (family member, relation, disease) from narratives; aims to structure anamnesis-derived FH for downstream use | NLP evaluation vs annotated corpus; precision/recall/F1 for entities/relations | Reported strong extraction performance (e.g., family-member F1 ≈0.88; family-side F1 ≈0.99; disease F1 ≈0.83; overall template accuracy reported) | Single-institution text patterns; FH only (not full anamnesis); portability across note styles uncertain | Family history (HNSCC risk, syndromic cancers) often buried in notes; structured FH can reduce missed hereditary signals and support risk stratification |
| Zakim, 2016 [23] | General medicine / patient intake | Conceptual + practical overview of automated history-taking ("medical interview" tools) and where they fit in care pathways | Narrative/clinical review; outcomes discussed include completeness, efficiency, usability | Frames automated anamnesis as a lever for time savings + standardization, but emphasizes design, safety, and workflow realities | Not an efficacy trial; heterogeneity of tools and contexts | Directly relevant to pre-visit H&N symptom capture (dysphagia, hoarseness, neck mass) and perioperative |

| | | | | | | |
|---|---|---|---|---|---|---|
| | | | | | | longitudinal intake |
| Denecke, 2018 [15] | General / self-anamnesis use case | Conversational user interface for self-anamnesis (rule-based dialog management) to collect history in a more natural interaction than forms | Prototype + usability-oriented evaluation | Demonstrated feasibility and user acceptance of conversational intake; highlights common failure modes in interpreting user input | Early prototype; limited clinical validation and domain breadth | Good precedent for conversational capture of symptom chronology/red flags before ENT–H&N visits |
| Zhang, 2018 [24] | EHR longitudinal data | Patient2Vec: interpretable deep representation of longitudinal EHR histories to enable personalized prediction | Model development/validation; predictive performance + interpretability | Shows longitudinal “history representations” can be both predictive and interpretable (attention-like mechanisms) | Not a patient-facing anamnesis tool; depends on EHR coding quality | Supports the logic that higher-quality structured histories (including patient-reported) can improve prediction in complex longitudinal H&N oncology pathways |
| Schwitzguébel, 2019 [25] | Ambulatory care | Automated history-taking device producing structured history to support differential diagnosis assessment | Clinical evaluation study (device-assisted history vs clinician workflow outcomes) | Demonstrates feasibility of automated HPI capture for DDx support in ambulatory settings | Device- and site-specific; generalizability; outcome definitions vary | Analogous workflow to H&N: structured symptom trajectories can support prioritization |

| | | | | | |
|---|---|---|---|---|---|
| | | | | | (e.g., malignant red flags) |
| Valmianski, 2021 [10] | Primary care pre-visit | SmartTriage: patient free-text “reason for visit” → ML classification; adaptive question sequencing using history; generates documentation + CDS outputs integrated with EMR | Retrospective development + system evaluation; metrics for CC prediction and downstream predictions | Demonstrates pipeline from patient intake to clinician-ready note + CDS; history improves predictions (especially in chronic/older patients) | Training labels partly derived from clinician notes; single system; not an RCT | Highly relevant architecture: pre-visit intake + adaptive questioning + documentation for ENT–H&N triage and clinic efficiency |
| Gashi, 2021 [26] | Digital health design | “Intelligent interviewers” lessons/guidelines for medical history collection (incl. conversational components) | Methods/guidance paper | Provides practical design guidance grounded in implementation experience | Not comparative clinical effectiveness | Helpful for building H&N-specific anamnesis agents (handling jargon, timeline, red flags, escalation rules) |
| Fukuzawa, 2022 [27] | Broad / cross-specialty | Review on the importance of patient history in AI-assisted diagnosis; emphasizes value of anamnesis features | Narrative review | Positions history as high-yield diagnostic signal for AI; motivates investment in better capture and representation | Not a clinical trial | Useful framing to justify H&N focus: symptom chronology and risk factors are decisive for pathway choice |
| Berdahl, 2022 [28] | Broad | Scoping review of digital tools to obtain HPI from patients (forms/conversational | Scoping review; outcome domains include usability, completeness, workflow effects | Shows heterogeneity of tools and limited high-quality | Evidence base uneven; outcomes nonstandardized | Directly supports argument for ENT–H&N- |

| | | | | | | |
|---|---|---|---|---|---|---|
| | | tools; outputs into clinical documentation) | | outcome evaluation; highlights recurring implementation barriers | | specific validation endpoints (missed red flags, time-to-diagnosis proxies) |
| Schmieding, 2022 [29] | Symptom checker apps (public-facing) | 5-year follow-up evaluation of symptom checker triage/diagnostic outputs using vignettes; compares 2015 vs 2020 | Vignette-based benchmarking; triage accuracy + diagnostic suggestion accuracy | Overall triage accuracy similar over time; changes in over/under-triage patterns; diagnostic and triage accuracy weakly correlated | Vignettes ≠ real patients; app scope constraints; versioning issues | Relevant to H&N safety: false reassurance for cancer red flags is a critical deployment risk; demands specialty-specific benchmarks |
| Albrink, 2022 [7] | Primary care | Protocol for assessing accuracy/usability of digital-device anamnesis vs clinician-led history | Study protocol; planned validity/usability outcomes | Defines rigorous framework for comparing patient-entered vs clinician history | Protocol only; results pending in that paper | Template for ENT–H&N: how to measure concordance on symptom onset, progression, smoking/alcohol, prior malignancy, radiation exposure |
| Scheder-Bieschin, 2022 [41] | German tertiary ED; real patients able to | Patient-facing AI symptom-taking tool (Bayesian/probabilistic reasoning with adaptive | Mixed-methods pilot observational (iterative V1→V2); feasibility, usability, perceived effects on | High patient-reported usability and perceived facilitation of | Small pilot; excludes higher-acuity/trauma; outcomes largely | Directly maps to H&N urgent pathways (e.g., dysphagia, neck |

| | | | | | | |
|---|---|---|---|---|---|---|
| | consent (pilot; 2 phases) | question flow) + HCP dashboard handover report | conversation/rapport, documentation, time efficiency | conversation; clinicians/nurses perceived utility, with time-saving felt more strongly by nurses than physicians | perceptual rather than clinical endpoints | mass, airway symptoms): pre-visit/triage intake + structured handover may improve urgency recognition and streamline documentation |
| Wallace, 2022 [8] | Digital/online symptom checkers evaluated on real or simulated patients across multiple medical conditions | Symptom checker tools that generate ranked differentials + triage advice from user-entered symptoms/demographics | Systematic review; diagnostic accuracy (primary diagnosis) and triage accuracy; quality assessment with QUADAS-2 | Across included studies, primary diagnostic accuracy was low (reported range ~19%–37.9%), while triage accuracy was higher but variable (~48.8%–90.1%) | Only 10 studies met inclusion; heterogeneous tools/conditions and evaluation methods; many vignette-based | High relevance for H&N safety: "red-flag" symptom patterns (neck mass, dysphagia, persistent dysphonia, otalgia) require conservative triage thresholds; emphasizes risk of false reassurance and need for specialty benchmarking |
| Hong, 2022 [51] | Family medicine clinic patients at a | Conversational AI "medical interviewing" system using an avatar + chatbot to capture | Feasibility/acceptability pilot; post-interview survey (Likert) on clarity, ease of use, attitudes, perceived usefulness | Most participants felt the system could help PCPs better understand | Small, highly educated sample; outcomes largely perceptual; no | Directly translatable to H&N pre-visit intake: |

| | | | | | | |
|---|---|---|---|---|---|---|
| | US academic center (pilot; n=20) | personal/family history and identify risk factors | | their health and identify risks; acceptance was generally positive but opinions varied on effort and appropriateness of AI interviewing | validation against clinician history or clinical endpoints | structured capture of risk factors (tobacco/alcohol/HPV exposure), symptom timelines, and escalation prompts before clinic |
| Kumar, 2022 [52] | Broad disease diagnosis literature (multiple diseases, modalities) | Systematic literature review of AI techniques for disease diagnosis (ML/DL across imaging and other data); includes PRISMA selection and synthesis framework | Systematic review (up to Oct 2020 searches); compares performance metrics reported across studies | Provides high-level mapping of AI methods, data types, and reported metrics; emphasizes breadth of applications and recurring challenges | Not focused on anamnesis or conversational systems; diagnostic-AI wide scope; limited direct applicability to history-taking workflows | Useful only as background framing: supports argument that diagnosis performance depends on data modality/quality—strengthening the case that high-fidelity history capture is a key input in H&N pathways |
| Badempet, 2023 [42] | Technical/preprint project; “disease prediction” focus | ML disease prediction (XGBoost on public datasets for diabetes/heart/liver) + chatbot layer (NLP / possibly OpenAI API) deployed on cloud | Engineering report; evaluation via standard ML metrics; no clinical validation for history-taking | Proposes integrated disease prediction + chatbot guidance; emphasizes scalability and | Preprint; not peer-reviewed; chatbot component described broadly; not focused on anamnesis quality or safety governance | Limited direct H&N value, but highlights a pattern you can critique: “diagnosis chatbot” |

| | | | | | | |
|---|---|---|---|---|---|---|
| | | | | deployment aspects | | without specialty-safe red-flag logic and validated intake is risky in oncologic symptom settings |
| Noack, 2023 [30] | Urgent care / primary care | Evaluation of an app for digital medical history taking prior to urgent care consultation | Prospective evaluation (app use, usability, feasibility, workflow outcomes) | Demonstrates feasibility and patient acceptance; highlights data quality considerations | Often single-center; outcome heterogeneity | Very relevant to urgent ENT–H&N access models (rapid triage for neck mass, airway/swallow symptoms) |
| Bouh, 2023 [31] | Health informatics | ML approach to digitize medical history and archive in a standard format | Technical development/evaluation | Demonstrates feasibility of standardizing stored anamnesis for reuse/interoperability | Often technical proof-of-concept; limited clinical evaluation | Supports H&N multidisciplinary continuity (tumor board, survivorship, perioperative teams) via reusable structured history |
| Zhu, 2023 [53] | AI-driven history-taking question selection (virtual care context) | Two-stage “retrieve + re-rank” history-taking: expert system retrieves candidate questions; dialogue-contextualized neural re-ranker (global re-ranker) selects best | ML4H methods paper; ranking metrics (nDCG, mAP) vs expert baseline and neural baselines | Proposed global re-ranker achieved substantial gains over expert system (reported +30% nDCG and +77% | Model-level evaluation; depends on available dialogue data; does not directly report patient outcomes or safety | Relevant for building H&N adaptive questioning that prioritizes red flags and compresses |

| | | | | | | |
|---|---|---|---|---|---|---|
| | | question; evaluates transformer vs S4 backbones | | mAP for best model) | | interview time (few high-yield questions early), especially for urgent referral triage |
| Zhakhina, 2023 [5] | Broad clinical settings (review perspective; pre-consultation workflows) | Review of pre-consultation history-taking systems (questionnaires/surveys completed before visits), discussing benefits (completeness, time efficiency, engagement), limitations (digital divide, data accuracy), and future role of AI/NLP for prioritization and personalization | Narrative review; outcomes discussed conceptually (efficiency, completeness, engagement, security) and illustrated with selected published examples | Argues that collecting histories pre-visit can improve completeness, shift clinician time toward targeted decision-making, and enhance engagement, while highlighting equity and security constraints | Not a systematic review; heterogeneous examples; no pooled effect estimates | Directly supports H&N implementation framing: pre-visit intake may reduce redundant questioning across multidisciplinary pathways, but digital inclusion and safe red-flag escalation must be designed explicitly |
| Gibelli, 2024 [1] | Ethical/legal domain across healthcare systems | Bioethical and medico-legal analysis of the shift from traditional to digital anamnesis; addresses narrative fidelity, confidentiality/privacy, doctor–patient relationship, communication barriers, and safety implications | Narrative review with historical overview of digital anamnesis development and discussion of governance concerns | Emphasizes that digital anamnesis may increase completeness and reduce workload but risks weakening clinician–patient dialogue; highlights privacy, | Not an effectiveness study; argumentation depends on cited literature and legal/ethical interpretation | Highly relevant to H&N surgery where histories can be sensitive (oncology, substance use) and delays are high-stakes; reinforces need for governance, |

| | | | | | | |
|---|---|---|---|---|---|---|
| | | for low digital/health literacy | | accountability, and safety issues in vulnerable populations | | consent, and clear responsibility for red-flag handling |
| Hindelang, 2024 [3] | Chatbots used for medical history-taking across fields/populations | Conversational agents collecting history (chatbots/virtual assistants) with focus on feasibility, usability, acceptance | Comprehensive systematic review (search through July 2024); includes observational studies and RCTs; quality appraisal (STROBE, RoB2) | Concludes chatbots can support engagement and streamline data capture; evidence quality mixed, with limited high-quality trials | Only English; excludes conference papers; heterogeneity of interventions/outcomes limits quantitative synthesis | Helps justify adoption questions in H&N surgery: acceptability, data security, and preservation of empathy during sensitive oncologic symptom discussions |
| Hauber, 2024 [6] | Outpatient cardiology | Tablet-based CAHT (rule-based dynamic questionnaire) generating narrative report integrated into practice management system | Prospective pilot; completion time, acceptance, documentation support | High completion, acceptable time, strong patient acceptance; report generation feasible; iterative improvement reduced errors | Single specialty/site; not LLM; outcomes mainly process/acceptance | Strong analogy: structured pre-visit capture could reduce redundancy and improve preparedness for complex H&N oncology follow-ups |
| Harada, 2024 [32] | Japan, symptom checker use | Longitudinal evaluation of diagnostic accuracy changes over time for an AI symptom checker | Observational/benchmarking; diagnostic accuracy vs time/version changes | Shows performance is not static; motivates "algorithmovigilan | Context-specific; evaluation approach varies | Crucial for H&N: safety monitoring for evolving tools (missed |

| | | | | | | |
|---|---|---|---|---|---|---|
| | | | | ce" and ongoing monitoring | | malignant red flags) is non-negotiable |
| Erden, 2024 [33] | Cardiology outpatient clinic | Study on how automatic history-taking software affects data quality | Clinical evaluation focused on documentation/data quality | Reports measurable effects on data quality and documentation workflow | Specialty-specific; generalizability uncertain | Data quality lens is directly transferable to H&N (accuracy of onset/duration, weight loss, dysphagia severity, prior RT/chemo) |
| Zagade, 2024 [43] | General-purpose disease prediction (user symptom input) | ML/NLP chatbot for symptom extraction → disease classification → preventive/hospitalization /medication guidance; dataset encoded in JSON | Development/implementation paper; no rigorous patient-facing history-taking validation described | Demonstrates a basic pipeline of symptom capture and disease prediction within a chatbot interface | Limited methodological detail; unclear ground truth, validation, and safety; not history-taking–specific | Useful as "lower-evidence" example: contrasts with clinically evaluated intake systems; underscores need for H&N-specific red-flag escalation and prospective validation |
| Sa, 2024 [44] | Primary clinical guidance (rural/after-hours access motivation) | "Robotic Medical Support ChatBot" using ML classification with predefined keyword extraction and class-label | Conference paper; experimental results described at a high level (classification efficacy) | Claims timely advice/temporary solutions and reduced need for immediate consultation | Rule-/keyword-heavy; unclear dataset and clinical evaluation; high risk of unsafe advice if | Again mainly a cautionary comparator: in H&N, advice-giving without validated triage |

| | | | | | | |
|---|---|---|---|---|---|---|
| | | mapping; outputs include first-aid medication info | | | used beyond intended scope | thresholds could delay cancer diagnosis or airway emergencies |
| Holderried, 2024 [45] | Medical education; 28 medical students | GPT-3.5 simulated patient chatbot guided by an illness script + behavioral prompts; focuses on history-taking dialogue realism | Prospective mixed methods; script adherence, plausibility of answers, usability (CUQ) | Most answers plausible; when questions covered by script, answers largely script-based; off-script questions often filled with fictitious details; overall CUQ positive | Hallucination/role drift still occurs; educational setting only; depends on script quality | Translationally relevant for H&N training: can create repeatable red-flag interviewing practice, but needs guardrails to prevent “plausible but wrong” symptom histories |
| Holderried, 2024 [46] | Medical education; 106 conversations; 1894 Q/A pairs | GPT-4 simulated patient + automated structured feedback on completeness of history taking; compared with human rater | Prospective study; human–AI agreement (Cohen κ), plausibility, feedback category performance | Very high plausibility (>99%); “almost perfect” agreement overall with human feedback (κ≈0.83), with weaker agreement in a subset of categories | Some feedback categories overly specific or diverging from human judgement; educational—not clinical—validation | High relevance for H&N training pipelines (ENT clinic/oncology): can standardize feedback for interviewing neck mass/dysphagia/voice change while flagging |

| | | | | | | |
|---|---|---|---|---|---|---|
| | | | | | | where specialty rubrics must be carefully encoded |
| Lepore, 2024 [47] | Dataset of 1213 medical histories; disease classes (cardiac, GI, thyroid) | Feature construction approach (Situation Awareness + Granular Computing) to improve ML classification of anamnesis; tested with multiple classifiers | ML experimental study; balanced accuracy across classifiers; improvement vs "traditional" feature sets | Balanced accuracy >90%; best around 93% (SVM); reported mean improvement ~16 percentage points with proposed feature construction | Not conversational; relies on existing histories; external validity depends on dataset representativeness | Useful methodological bridge: shows that how you represent history strongly affects downstream classification—central for H&N where symptom chronology and risk factors drive pathway selection |
| Larsson, 2025 [9] | Swedish primary care | Qualitative study of integrating AI-based triage using Normalization Process Theory | Interviews/qualitative analysis; implementation determinants | Adoption depends on workflow integration, responsibility clarity (red flags), interoperability, perceived usefulness | Context-specific; not an accuracy study | Directly maps to ENT–H&N: clear escalation rules and medico-legal responsibility for "red flag" outputs are essential |
| Ilicki, 2025 [34] | Digital triage safety | Symptom checker evaluation using real-world vignettes anchored | Vignette validation; triage accuracy/safety lens | Emphasizes safety-critical benchmarking | Vignettes; tool/version specificity | Highly relevant: H&N delayed cancer diagnosis is a |

| | | | | | | |
|---|---|---|---|---|---|---|
| | | to historical triage-related adverse events | | grounded in adverse events | | classic adverse-event class; methodology could be adapted |
| Rädel-Ablass, 2025 [35] | Medical education | AI-based role plays for anamnesis teaching; examines teaching opportunities and acceptance | Educational evaluation; acceptability/learning perceptions | Supports feasibility of AI role-play for history-taking training | Educational endpoints; not clinical outcomes | Could train ENT–H&N red-flag interviewing and difficult conversations (e.g., smoking/alcohol, symptom progression) |
| Yuan, 2025 [37] | Stomatology clerkship | AI agent acting as simulated patient for history-taking training | Educational feasibility study | Shows simulated patient agents can scaffold training and standardize exposure | Domain-specific; generalization uncertain | Bridge to oral cancer screening/anamnesis training within head & neck oncology pathways |
| Wang, 2025 [38] | Medical education | GPT-based history-taking training tool; feasibility study | Educational trial/feasibility outcomes | Suggests feasibility and learner engagement; highlights need for safeguards against incorrect feedback | Education-focused; tool/version dependence | Potential for structured rehearsal of H&N symptom elicitation and risk-factor interviewing |
| Laverde, 2025 [36] | Virtual patient training | LLM-based agents integrated into virtual patient chatbot for history-taking training | System development + evaluation | Demonstrates richer dialog + automated feedback potential | Safety/ground-truthing of feedback remains key | Could support H&N-specific simulated cases (neck mass |

| | | | | | | |
|---|---|---|---|---|---|---|
| | | | | with LLM-based agents | | workup, dysphagia pathway) with standardized feedback |
| Samojluk, 2025 [39] | Voice-based interviewing | Prototype voice interview system supporting diagnosis | Prototype development; feasibility/performance | Demonstrates feasibility of voice anamnesis capture for diagnostic support | Prototype; limited clinical validation | Voice interfaces may benefit H&N populations with limitations (elderly, low literacy), but must handle dysphonia/dysarthria robustly |
| Umerenkov, 2025 [11] | Large-scale EHR/CDSS | AIDA: predictive model for ICD diagnosis from health records; highlights importance of textual history fields | Retrospective validation + deployment metrics | High accuracy vs clinicians; history fields highly informative; uses confidence gating | Local data; limited external validation | Supports integrating structured patient-reported histories into decision support for complex H&N oncology care |
| Park, 2025 [14] | Emergency medicine (simulated) | ChatGPT vs physicians; evaluates history taking, clinical accuracy, communication | Simulated patient study | ChatGPT scored higher in history-taking/communication; accuracy comparable in simulation | Simulated cases; deployment safety unresolved | Reinforces opportunity (better history capture) but flags need for governance in high-risk H&N red-flag contexts |

| | | | | | | |
|---|---|---|---|---|---|---|
| Tu, 2025 [13] | OSCE-like evaluation | AMIE conversational diagnostic AI optimized for dialog | Randomized, blinded OSCE-style comparisons with clinicians | Strong performance across information gathering/diagnostic axes in controlled setting | Text-only OSCE; real-world safety unknown | Suggests upper bound for conversational systems; H&N needs domain constraints + escalation + safety monitoring |
| Lembo, 2025 [19] | Rare disease diagnostic process | AI4RDD framework: multimodal intake + symptom analysis + physician hypotheses + ranked diagnosis; HDKS concept | Framework/architecture proposal + PoC latency | Provides blueprint for holistic anamnesis + multimodal integration + governance | Not clinical effectiveness; RD focus | Architectural inspiration for complex H&N pathways (multimodal reports/imaging + MDT notes + patient-reported outcomes) |
| Chappidi, 2025 [40] | EHR endpoints | Compares outcome endpoints derived from diverse EHR modalities; relates to how "history" is operationalized | Methods paper | Shows endpoints vary across modalities and can change model conclusions | Not an anamnesis tool; methodological | Important for H&N outcomes research: how you encode history affects inference and AI evaluation |
| Narsimhulu, 2025 [48] | General "digital health partner" concept; patient-facing assistant | Chatbot with NLP + RAG/LLM + FAISS (PDF ingestion via PyPDF2) + Streamlit UI; multilingual support, appointment scheduling, medication reminders | Technical/descriptive article; no clinical validation of anamnesis capture reported | Emphasizes architecture and features (RAG, scheduling, reminders); positions as health-care access tool | Low methodological rigor; unclear evaluation; broad claims; privacy invoked but not demonstrated | Relevant as a design pattern to critique/adapt: H&N could benefit from patient education + |

| | | | | | | |
|---|---|---|---|---|---|---|
| | | | | | | navigation + pre-visit intake, but only if safety/triage logic and data governance are robust |
| Yi, 2025 [49] | Medical education; Korean virtual patient; 5 expert reviewers | Virtual patient using generative AI (HyperCLOVA X) for history-taking practice; dialogue scripts analyzed for plausibility | Pilot feasibility; Likert ratings (relevance/validity/accuracy/succinctness/fluency) + discourse analysis | Small proportion of implausible words (~2.6%); experts rated relevance/validity/accuracy relatively high; fluency more mixed | Very small pilot; single case; language/cultural context; education-only | H&N adaptation potential: language-specific VPs for interviewing culturally sensitive risks (tobacco/alcohol/HPV) and red-flag symptom patterns |
| Liu, 2025 [50] | Medical education; 31 students; 3 cases (simple→complex) | AMTES: LLM-based virtual SP (DeepSeek-V2.5) + automated evaluation framework; tested also with Qwen-Max (generalizability) | Prospective multicase study; dialog accuracy/context appropriateness; evaluation stability (CV), human–AI consistency (ICC), transparency | High dialog accuracy (~98–99%); strong evaluation stability (low CV); ICCs >0.923; high item-level consistency; students reported high usefulness | Education setting; depends on rubric design; still requires governance against hallucinations/overconfidence | Very relevant as a “training + assessment” blueprint for H&N: multicase scenarios (neck mass, progressive dysphagia, unilateral otalgia) and rubric-based evaluation could be |

| | | | | | | |
|---|---|---|---|---|---|---|
| | | | | | | implemented for residency/fellowship training |
| Gehlen, 2025 [54] | Orthopedics/trauma vignettes (30 cases); 4 specialists vs 4 symptom checker apps | Symptom checker chatbots compared with physicians for diagnostic accuracy and urgency assessment | Experimental multi-observer vignette study; % correct diagnosis and urgency; correlations (Spearman) | Physicians outperformed apps (diagnosis ~84% vs ~36%; urgency ~70% vs ~21%, as reported) | Vignettes; orthopedics only; app selection/versioning affects results | Strong analog for H&N: reinforces that current general symptom checkers may underperform on urgency—critical in airway compromise or cancer suspicion pathways |
| Siira, 2025 [2] | Empirical research on AI systems automating history taking and triage (86 studies) | Scoping review mapping AI systems (tasks, maturity/readiness, stakeholder perspectives, implementation barriers) | PRISMA-ScR scoping review; databases include PubMed, CINAHL, PsycINFO, Scopus, Web of Science | Most studies were prototypes/retrospective; very few evaluated real-time clinical integration or stakeholder perspectives; barriers grouped into technical, context/culture, end-user engagement, evaluation process | Scoping nature; heterogeneity; many studies lacked methodological clarity | Very useful for H&N framing: highlights translational gaps—need for prospective integration studies, stakeholder buy-in, and governance for red-flag responsibility and interoperability |

| | | | | | | |
|---|---|---|---|---|---|---|
| Craamer, 2025 [4] | CAHT in elective and acute care (19 studies; ~11,885 patients) | Computer-assisted history taking (digital questionnaires/medical interviews) completed pre-visit; emphasis on workflow/EHR integration | Systematic review (PRISMA); outcomes: completion rates, satisfaction, engagement, efficiency, consultation duration, diagnostic accuracy | Completion rates generally high; satisfaction consistently high; suggests gains in engagement and workflow/resource use; mixed effects on visit duration; some evidence for improved diagnostic alignment through comprehensive capture | Evidence heterogeneous; implementation and EHR integration variable; digital inclusion concerns | Directly applicable to H&N clinic flow: pre-consult CAHT can standardize symptom chronology and risk-factor capture, reduce redundant questioning across MDT pathways |
| Laurent, 2025 [55] | Industry/web overview across multiple chatbot types | Narrative “10 key examples” of healthcare chatbots (triage, chronic care, mental health, etc.), mixing evidence claims and product descriptions | Non–peer-reviewed web review; summarizes example systems and selected studies | Useful taxonomy of chatbot roles and common claims; highlights widespread adoption narratives | Not a scientific study; selective evidence; unclear methods and bias control | Best used sparingly (or in a background box) to describe the broader ecosystem that H&N patients may already encounter outside clinical governance |
| Author, year (Ref) | Clinical context / population | System & AI approach (single-cell summary) | Study design & outcomes assessed | Main findings | Key limitations / notes | Potential relevance to head & neck surgery |
| Madda, 2025 [56] | Standardized clinical vignettes | Adaptive-questioning symptom checker (CareRoute) tested | Interactive vignette-based evaluation; primary outcomes: triage concordance + elicitation | Exact 3-tier triage concordance 88.9% (40/45); no | Preprint, not peer-reviewed; single physician evaluator; | Methods are directly portable for |

| | | | | | | |
|---|---|---|---|---|---|---|
| | (Semigran BMJ set; 45 cases) | interactively starting from presenting complaint only; history-taking quality quantified via normalized feature elicitation metrics; outputs triage recommendations | coverage/fraction; secondary: user burden (questions/time) | under-triage on emergency-reference vignettes; median elicitation coverage 67% and elicitation fraction 70%; urgency-conditioned questioning (fewer questions for emergency cases) | vignette-based (not real patients); conservative triage mapping (4-tier to 3-tier) | H&N benchmarking: you could build H&N red-flag vignettes (neck mass, dysphagia, persistent dysphonia, otalgia) and measure both triage safety and history elicitation quality rather than only final recommendations |
